# Indigenous children and adolescent mortality inequity in Brazil: What can we learn from the 2010 National Demographic Census?

**DOI:** 10.1016/j.ssmph.2020.100537

**Published:** 2020-01-09

**Authors:** Ricardo Ventura Santos, Gabriel Mendes Borges, Marden Barbosa de Campos, Bernardo Lanza Queiroz, Carlos E.A. Coimbra, James R. Welch

**Affiliations:** a Escola Nacional de Saúde Pública, Fundação Oswaldo Cruz, Rua Leopoldo Bulhões 1480, Rio de Janeiro, RJ, 21041-210, Brazil; b Departamento de Antropologia, Museu Nacional, Universidade Federal do Rio de Janeiro, Quinta da Boa Vista s/n, Rio de Janeiro, RJ, 20940-040, Brazil; c Instituto Brasileiro de Geografia e Estatística, Av. Presidente Antonio Carlos 25, Rio de Janeiro, RJ, 20020-010, Brazil; d Departamento de Sociologia, Faculdade de Filosofia e Ciências Humanas, Universidade Federal de Minas Gerais, Av. Antônio Carlos 6627, Belo Horizonte, MG, 31270-901, Brazil; e Centro de Desenvolvimento e Planejamento Regional, Faculdade de Ciências Econômicas, Universidade Federal de Minas Gerais, Avenida Antônio Carlos 6627, Belo Horizonte, MG, 31270-901, Brazil

**Keywords:** Child mortality, Adolescent mortality, Ethnicity, Indigenous peoples, Health disparity

## Abstract

Indigenous peoples worldwide are highly disadvantaged compared to national baseline populations. Given historical challenges to accessing relevant data for Brazil, the present study innovates by using 2010 Brazilian National Demographic Census data to estimate mortality curves in Indigenous children and adolescents <20 years. The non-parametric smoothing approach TOPALS (tool for projecting age-specific rates using linear splines) was employed. Analyses included stratifications by sex, rural or urban residence, and geopolitical region. The mortality of children and adolescents classified as Indigenous was higher for all analyzed strata. Mortality of Indigenous and non-Indigenous individuals in rural areas was higher than those in urban areas in almost all strata analyzed. Mortality levels in the Indigenous segment exceed those of children and adolescents classified as non-Indigenous in all four geopolitical regions, with few exceptions. This is the first study to compare mortality curves of children and adolescents in Brazil according to social variables based on national census data. More Indigenous children and adolescents die than their non-Indigenous counterparts, including those classified as black or brown, in both rural and urban residential settings. Indigenous children and adolescents are consistently at the most disadvantaged end of a marked gradient of ethnic-racial inequality in Brazil, independently of sex, age, and geopolitical region.

## 1 Introduction

Indigenous peoples worldwide are highly disadvantaged in terms of access to education, housing and sanitation, food security, and morbidity and mortality rates (Carson, Sharmin, Maier, & Meij, 2018; I. Anderson et al., 2016; King, Smith, & Gracey, 2009; Stephens, Porter, Nettleton, & Willis, 2006). These peoples’ similarly violent histories of colonization contribute to experiences of extreme marginalization, including many challenges in retaining access to ancestral lands and maintaining their cultures and traditions, which are shown to impact health and wellbeing in a multitude of ways (I. Anderson et al., 2016; Mitrou et al., 2014; Stephens et al., 2006).

Growing recognition of major health gaps between Indigenous and non-Indigenous populations has led various countries to work towards implementing specific policies aimed at reducing inequities. International agencies and nongovernmental organizations have also developed agendas to debate and propose policies to reduce Indigenous and non-Indigenous health inequity (United Nations, 2009). Yet, in most countries, Indigenous peoples are seldom counted as separate social categories by national demographic censuses and health information systems, producing statistical invisibility with potentially long-lasting political implications for entire populations. Basic health statistics about Indigenous populations, such as morbidity and mortality, are often calculated only through manual, meticulous, or costly extraction of data from national vital records or health information systems (I. Anderson et al., 2016). Only in a few countries, such as Canada, the United States, New Zealand, and Australia, have quality data from birth and mortality registries and population-based epidemiological studies become systematically available in recent decades, thereby facilitating the national characterization of Indigenous peoples’ health inequities (R. N. Anderson, Copeland, & Hayes, 2014; Mitrou et al., 2014; Tobias, Blakely, Matheson, Rasanathan, & Atkinson, 2009).

Analyses of Indigenous peoples’ health in Latin America have long been impeded by low coverage and deficient quality of population registries, as well as difficulties associated with ethnic and racial identification from health information sources (Loveman, 2014; Montenegro & Stephens, 2006). In numerous countries, the absence of specific categories used to identify Indigenous groups in official statistics has been attributed to the historical presumption that they would soon be assimilated into dominant national populations and cease to exist as socioculturally differentiated peoples (Santos et al., 2019). More recently, academic studies addressing health inequities among ethnic and racial groups have expanded markedly and thereby influenced public policy formulation and implementation aimed at reducing inequities in many Latin American countries (Montenegro & Stephens, 2006). The Brazilian case is illustrative, with demographic and health information sources about the Indigenous population having increased since the 1990s, principally after the national census and the principal health information systems began collecting data using the category “Indigenous” (*indígena*) (Santos et al., 2019). Beginning in 2010, the Brazilian national census became an even more important tool for analyzing health indicators by introducing a specific question about the occurrence of deaths in households during the prior year.

Child mortality has long been widely recognized as a synthetic variable capturing population-wide quality of life and specific health conditions of children under 1 year (Victora et al., 2003). Recently, interest has increased in analyses of mortality among children over 5 and adolescents under 20 years due to, among other reasons, the global youthening of the nutrition transition and increase in causes of mortality associated with mental health and violence in this age group (Masquelier et al., 2018; Nair & Byass, 2018).

In Brazil, two recent studies analyzed ethnic and racial mortality inequalities based on the 2010 Brazilian census. Caldas et al. (2017) reported significantly higher Indigenous infant mortality rates than those observed in the general Brazilian population (27.3 and 15.9 deaths per thousand live births, respectively). Campos, Borges, Queiroz, and Santos (2017) estimated Indigenous mortality to be approximately double that of the non-Indigenous population among children in the age groups 0–4.9 and 5.0–14.9 years.

Given the historical challenges to accessing relevant data for Brazil and increased interest in child and adolescent mortality worldwide, the present study innovates by using Brazilian census data to estimate mortality curves in Indigenous children and adolescents to better understand the country's racial and ethnic health and social disparities. The object of the study is to compare mortality levels of Indigenous and non-Indigenous children and adolescents under 20 years in Brazil. This is the first study to use Brazilian census data to compare inequality in mortality rates during the first two decades of life according to social variables, including race or color classification, urbanization, and geopolitical region.

## 2 Methods

To estimate mortality of Indigenous children and adolescents (i.e., individuals < 20 years), this paper uses data on household mortality derived from the 2010 Brazilian National Demographic Census, which is the most recent census undertaken in the country. Census interviewers asked about the occurrence of deaths of residents in the previous 12 months in all 51,204,587 households in Brazil (excluding institutional households). Data on sex and age of the deceased were also collected. Using standard demographic methods proposed by Hill, You, and Choi (2009), Queiroz and Sawyer (2012) reported that household death data from the 2010 Census were of good quality regarding declaration of age and national coverage.

The Brazilian census collects information about skin color or race of respondents, using the following categories: white, black, brown (*pardo*), yellow, and Indigenous (Petruccelli & Saboia, 2013; Telles, 2004). This classification system has been in use since 1940, except for the category Indigenous, which was only included since 1991. An important innovation in the 2010 Census was the application of the question about color or race to all households and all persons, rather than to a sample. This point is particularly relevant for Indigenous peoples since this population segment represents only a small proportion of the total Brazilian population. In 2010, the color or race composition of the Brazilian population was 47.7% white, 7.6% black, 1.1% yellow, 43.1% brown, and 0.4% Indigenous. For the purposes of this study, the category yellow was excluded due to recent studies showing irregularities in how this term was registered in national surveys (Petruccelli & Saboia, 2013).

It is noteworthy that in the 2010 Census, no information was collected on the color or race of the individual who died. Attending to the present study's focus on the Indigenous population, different classification typologies of Indigenous households were compared: (1) households with at least one Indigenous resident; (2) households with more than half Indigenous residents; (3) households with all Indigenous residents; (4) head of household classified as Indigenous. Mortality estimates were similar for all four alternatives (results not shown), so we chose to use the method described in item 4, whereby the individual who died was attributed to the same color or race category as the head of household.

The high stochastic variation present in a relatively rare phenomenon such as death poses a challenge for mortality estimations for small population groups (Alexander, Zagheni, & Barbieri, 2017). Demographic events such as births and deaths, even when derived from a complete census or vital statistics systems, are subject to random variation and may be assumed to follow a Poisson distribution, as proposed by Brillinger (1986). Thus, let the number of deaths $D_{\alpha }$ for population group α be Poisson distributed as follows:

$$D_{\alpha }\;\sim \;Poisson\left(P_{\alpha }\cdot h_{\alpha }\right)$$

where $P_{\alpha }$ is the population exposed to the risk of dying and $h_{\alpha }$ is the mortality rate for group the population group α. In this paper, α refers to a certain group of color/race, region, urban/rural condition, sex, and age.

Parametric and non-parametric methods may be used to smooth noisy mortality rates and capture their main regularities throughout different age groups. The demographic analysis carried out in this study is based on the non-parametric smoothing approach using the TOPALS relational model proposed by Gonzaga and Schmertmann (2016), which is a variant of the original one developed by de Beer (2012). TOPALS uses linear spline to model the risk ratio function that relates a vector of observed age-specific mortality rates $h_{\alpha }$ of a given group with the standard mortality schedule $h_{\alpha }^{std}$. Mortality age profiles for all sub-populations investigated in the present study were estimated using as standards the national profile derived from the official life table published by the Brazilian Institute of Geography and Statistics (IBGE) (2012).

The model was fitted using the software R. The R script utilized in this paper were adapted from material available in the GitHub repository, available at https://github.com/schmert/TOPALS.

In addition to comparing Indigenous mortality curves with the other color or race groups used in official statistics in Brazil, analyses also included stratifications by sex, rural or urban residence, and geopolitical region. It should be emphasized that the rural/urban distribution of the Indigenous population in Brazil (61.5% rural) is quite different from that of the non-Indigenous segment (only 15.4% rural). For analysis by geopolitical region, the country's five major regions identified by the IBGE were recombined into four (Central-West, North, Northeast, South/Southeast) due to smaller Indigenous populations in the South and Southeast. The largest proportion of Indigenous people (37.4%) is concentrated in the North region, while the largest proportion of non-Indigenous people (56.6%) is located in the South/Southeast (source: www.sidra.ibge.gov.br, accessed July 19, 2019).

## 3 Results

In total, 76.099 deaths of individuals <20 years were reported in the 2010 census (34.6% white, 12.8% black, 51.2% brown, and 1.4% Indigenous). Proportionally, these values were more elevated for those classified as black, brown, and Indigenous when compared to the overall distribution of people <20 years in the country by color or race of the head of the household (41.3% white, 10.3% black, 47.7% brown, and 0.7% Indigenous).

The distribution of the Indigenous Brazilian population <20 years of age according geopolitical region of residence shows the proportion of residents in rural areas is high (74.5%), ranging from 54.4% in the South/Southeast to 84.7% in the Central-West (Table 1).

**Table 1:** Distribution of Indigenous Brazilian population <20 years of age, according to geopolitical region and rural or urban residence, 2010 Brazilian Demographic Census.

Region	Rural	Urban	Total
North	141,015 (83.9%)	27,092 (16.1%)	168,107 (100%)
Northeast	51,613 (60.3%)	33,910 (39.7%)	85,523 (100%)
South/Southeast	31,675 (54.4%)	26,527 (45.6%)	58,202 (100%)
Central-West	56,367 (84.7%)	10,163 (15.3%)	66,530 (100%)
Total	280,670 (74.2%)	97,692 (25.8%)	378,362 (100%)

**Fig. 1:**
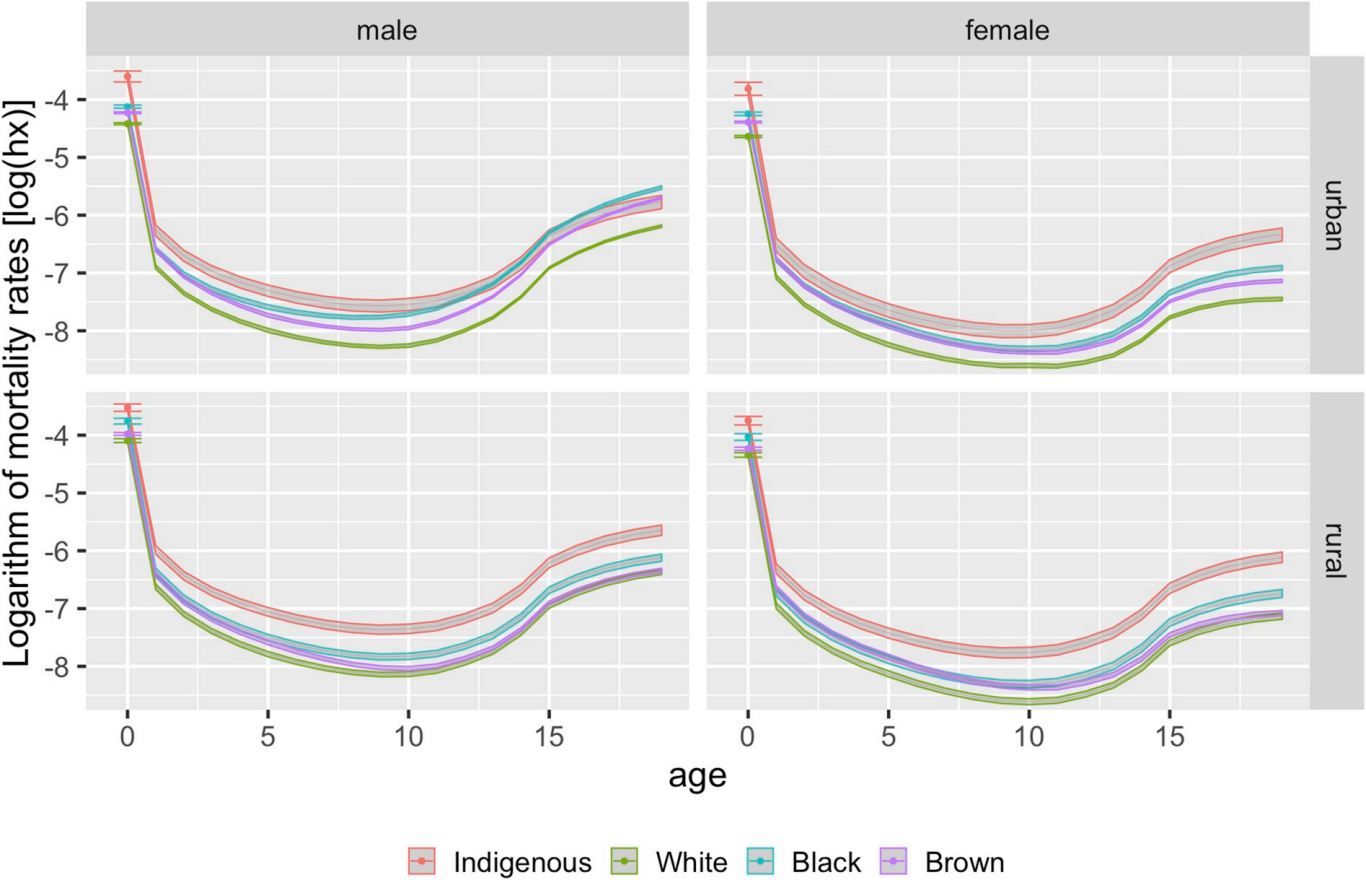
Mortality estimates (log_10_) and associated 95% confidence intervals in the Brazilian population < 20 years of age, according to sex, color or race, and urban or rural residence, 2010 Brazilian Demographic Census. (For interpretation of the references to color in this figure legend, the reader is referred to the Web version of this article.)

Fig. 1 shows mortality estimate curves with associated confidence intervals by age, sex, color or race, and urban or rural residence. Point estimates and confidence intervals for infant mortality are highlighted due to the relevance of this indicator for public health. Even considering the larger confidence intervals for the Indigenous population resulting from its smaller size, the mortality of children and adolescents classified as Indigenous was higher for all analyzed strata, particularly when compared to those classified as white. In general, the curves for children and adolescents classified as black and brown were between those of the Indigenous and white populations. One exception was the urban male, for whom the curves of Indigenous, black, and brown individuals overlapped from around 10–15 years of age. For all strata, whereas the curves of black and brown individuals converged for children <5 years, they diverged during the adolescent years, especially from age 10, with those classified as black being more elevated and those classified as brown being closer to the white population.

**Fig. 2:**
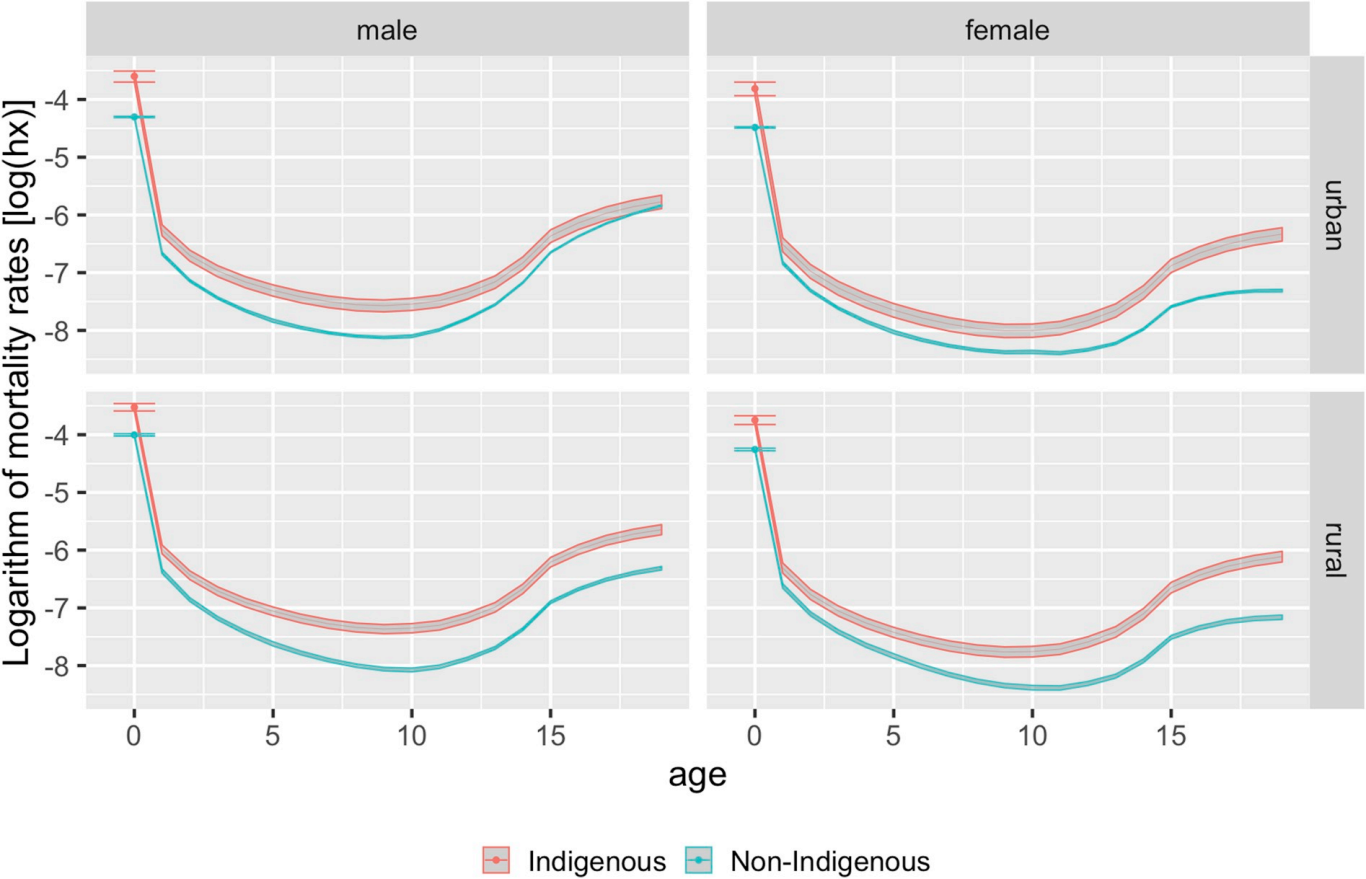
Mortality estimates (log_10_) and associated 95% confidence intervals in Indigenous and non-Indigenous Brazilian population < 20 years of age, according to sex and urban or rural residence, 2010 Brazilian Demographic Census.

The higher levels of mortality observed for the Indigenous category become even more evident when compared to the entire non-Indigenous population (white, black, and brown categories combined) (Fig. 2). The differences are striking, with virtually no overlap of the curves for either urban or rural residents. The only overlapping observed was for Indigenous and non-Indigenous men aged 19–20 living in urban areas.

Table 2 presents mortality estimates and their 95% confidence intervals (C.I.) comparing Indigenous and non-Indigenous categories in four age groups (0–0.9, 1–4.9, 5–9.9, and 10–19.9 years). In all analyzed strata, the mortality of Indigenous peoples was higher than the non-Indigenous segment, with no overlap in the 95% C.I. In all comparisons, mortality estimates were higher by at least half for Indigenous peoples, with few exceptions (female, urban and rural, 1–4.9 years; female, urban, 5–9.9 years; and male, urban, 10–19.9 years). In both the Indigenous and non-Indigenous populations, mortality rates were generally more than one-fourth higher among males than females in all age groups. In the age group 10–19.9 years, the mortality rate was three times higher among urban non-Indigenous males. Mortality of Indigenous and non-indigenous individuals in rural areas was higher than those in rural areas in all strata analyzed, except for non-Indigenous males in the age group 10–19.9 years.

**Table 2:** Mortality estimates and 95% confidence intervals (C.I.) in Indigenous and non-Indigenous Brazilian population < 20 years of age, according to age groups, sex and urban or rural residence, 2010 Brazilian Demographic Census.

Age group (years)	Sex	Urban/rural	Probability of dying (per 1000) (95% C.I.)	
			Non-indigenous	Indigenous
0–0.9	Male	Urban	13.4 (13.3; 13.6)	27.1 (24.6; 29.6)
		Rural	18.1 (18.7; 18.4)	29.1 (27.3; 30.9)
	Female	Urban	11.2 (11.1; 11.3)	21.9 (19.4; 24.5)
		Rural	14.1 (13.8; 14.4)	23.3 (21.6; 25.1)
1–4.9	Male	Urban	3.1 (3.1; 3.2)	4.9 (4.4; 5.3)
		Rural	4.1 (4.0; 4.3)	6.3 (5.9; 6.8)
	Female	Urban	2.6 (2.6; 2.7)	3.7 (3.3; 4.2)
		Rural	3.2 (3.1; 3.3)	4.5 (4.2; 4.9)
5–9.9	Male	Urban	1.7 (1.6; 1.7)	2.9 (2.6; 3.2)
		Rural	1.9 (1.9; 2.0)	3.6 (3.3; 3.9)
	Female	Urban	1.3 (1.3; 1.4)	2.0 (1.7; 2.2)
		Rural	1.5 (1.5; 1.6)	2.5 (2.2; 2.7)
10–19.9	Male	Urban	12.9 (12.7; 13.0)	15.9 (14.2; 17.7)
		Rural	9.3 (9.1; 9.5)	18.4 (16.9; 19.9)
	Female	Urban	4.4 (4.4; 4.4)	9.4 (8.3; 10.6)
		Rural	4.8 (4.6; 4.9)	11.8 (10.7; 12.9)

**Fig. 3:**
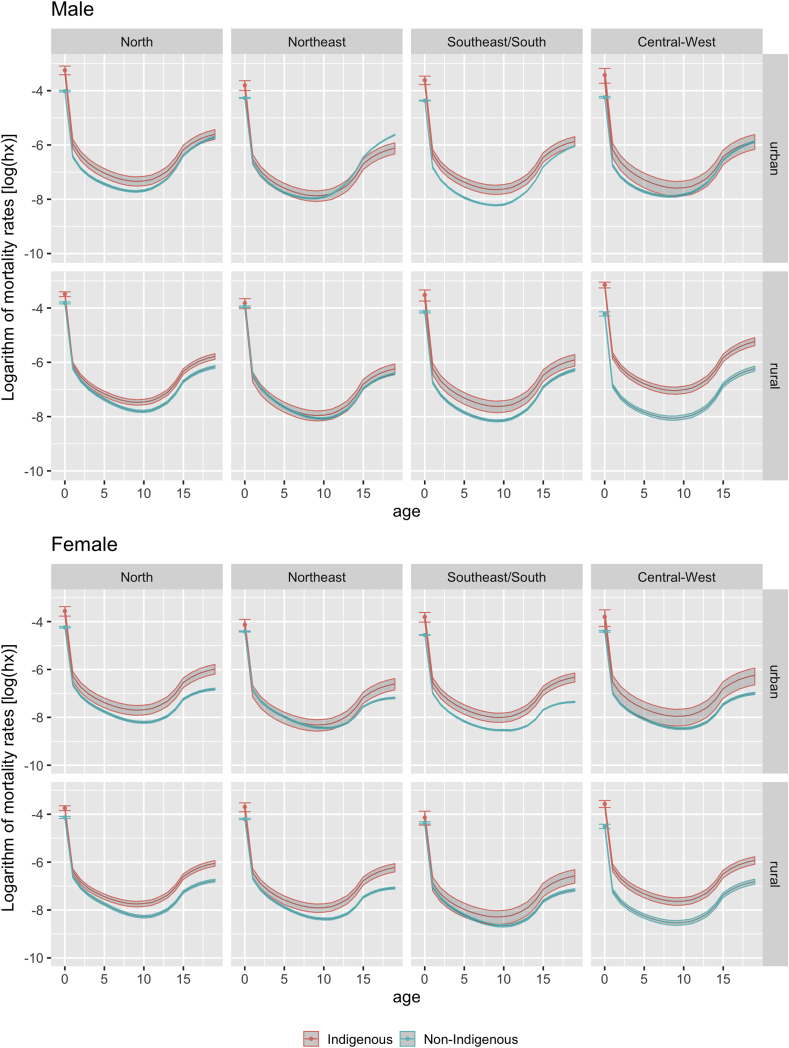
Mortality estimates (log_10_) and associated 95% confidence intervals in Indigenous and non-Indigenous Brazilian population < 20 years of age, by region and urban or rural residence, 2010 Brazilian Demographic Census.

Finally, Fig. 3 compares mortality curves between Indigenous and non-Indigenous categories by sex, urban or rural residence, and geopolitical region. Mortality levels in the Indigenous segment exceeded those of children and adolescents classified as non-Indigenous in all four regions, with few exceptions. Of the 16 comparisons presented, approximately two-thirds of the distributions were clearly differentiated, with the Indigenous segment presenting higher mortality levels.

## 4 Discussion and conclusions

The availability of health statistics is essential for understanding local, regional, and global socioeconomic and health inequities affecting ethnic Indigenous minorities (Carson et al., 2018; I. Anderson et al., 2016; Montenegro & Stephens, 2006; Santos et al., 2019). In the article by Setel et al. with the memorable title “A scandal of invisibility: making everyone count by counting everyone”, published in *The Lancet* a decade ago, the authors called attention to the fact that in many world regions a significant proportion of people are “born and die without leaving a trace in any legal record or official statistic … [rendering] most of the world's poor as unseen, uncountable, and hence uncounted” (Setel et al., 2007) (p. 1570). The multidimensional implications of this invisibility include hampering the formulation, implementation, and evaluation of policies aiming to reduce social inequities. The authors call attention to the need to improve civil registration systems, both in national and international settings, independently of race, color, or ethnicity, as well as to undertake population-based studies to generate reliable basic statistics and sociodemographic and health indicators.

Although there is evidence from various parts of the world that mortality rates are higher for Indigenous than for non-Indigenous populations, there remains a lack of demographic and epidemiological data that effectively characterizes such inequities for most countries (Gracey & King, 2009; Montenegro & Stephens, 2006; United Nations, 2009). In order to address this gap, Anderson et al. (2016) comparatively analyzed a comprehensive set of sociodemographic and health indicators representative of twenty-three countries, including Brazil. They demonstrated that the worst health indicators were concentrated in the Indigenous segments of the national populations of analyzed countries. The results of the present study, focusing on the mortality of Indigenous children and adolescents in Brazil, are in line with the evidence presented by Anderson et al. (2016) of marked inequities when comparing Indigenous to non-Indigenous national populations.

Despite advances and improvements in the coverage of demographic and health information systems for the Brazilian population in recent decades, incorporation of the Indigenous contingent, both rural and urban, remains deficient (Santos et al., 2019). Problems of under-registration of vital data including births and deaths are accompanied by the exclusion of Indigenous peoples from a substantial portion of the major epidemiological and demographic investigations of reference for Brazil.

The lack of information about Indigenous peoples has slowly and imperfectly improved in Brazil over the last several decades due to advanced analyzes based on national health information systems, demographic censuses, and a few population-based surveys (Caldas et al., 2017; Cardoso, Santos, & Coimbra Jr, 2005; Coimbra Jr. et al., 2013). Several of these studies have focused on children under the age of five. The general picture that emerges is that health indicators are consistently worse for Indigenous than non-Indigenous children. For example, the First National Survey of Indigenous People's Health and Nutrition in Brazil showed that Indigenous children are much more vulnerable to undernutrition and preventable infectious and parasitic diseases in primary care settings than the national reference population. According to this study, the prevalence of chronic undernutrition in Indigenous children under five was 25.7%, substantially higher than the 7.1% reported for children of the same age in the general Brazilian population (Coimbra Jr. et al., 2013).

The overall mortality profile in Brazil has changed rapidly, with a substantial reduction in child mortality between 1990 and 2012 from 47.1 to 14.6 deaths per 1000 live births (Ministério da Saúde, 2014). Contrary to this national trend, a recent study reported an Indigenous child mortality rate similar to the baseline national population decades earlier (Caldas et al., 2017). Although census data do not permit characterization of the specific causes of death, nationally representative studies show that Indigenous households in both rural and urban settings have less favorable levels of basic sanitation (Coimbra Jr. et al., 2013; Raupp, Fávaro, Cunha, & Santos, 2017), which potentially are associated with the higher levels of mortality observed in Indigenous children.

Whereas the health conditions of Indigenous children under the age of five have been the focus of extensive recent research efforts in Brazil, the scenario for children and adolescents from five to 20 years remains much less well known. To date, no specific Brazilian national surveys have been conducted to characterize the socioeconomic and health conditions of Indigenous children in this age group. Despite this information gap, several topics have been addressed more robustly in regional studies for late childhood and adolescent age groups. For example, child suicide has drawn special attention because Indigenous mortality rates are much higher than those reported for the general Brazilian population. Among children from 10 to 14 years, the suicide mortality rate from 2010 to 2014 was 11.0/100 thousand, 18.5 times higher than among non-Indigenous children in the same age group (0,6/100 mil) (Souza, 2019).

The present study is the first that compares mortality curves of children and adolescents in Brazil according to color or race categories and other social variables based on national census data. Our approach using 2010 census data allowed stratification by urban and rural residence, which is important because more than half of the self-declared Indigenous population in the 2010 Census lived in rural areas (Table 1) and other sources of data, such as the Brazilian national mortality information system, does not allow urban-rural disaggregation of death records (Caldas et al., 2017; Santos et al., 2019).

The 2010 Census data presents some limitations for characterizing mortality levels. Queiroz and Sawyer (2012) argue that data derived from the question about occurrence of deaths in households in the prior year could result in underestimation of mortality in single person households in which the resident died. In such cases, the household would no longer exist and therefore would not be interviewed, and the death would not be counted. This methodological question is particularly pronounced for households inhabited by elderly individuals. Since the analytical focus of the present study is the population under 20 years, this question potentially does not have a substantial impact on mortality estimates.

Brazil is well known internationally for presenting extreme polarization in indicators of social inequities, such as income levels, education, and sanitation (Szwarcwald, Souza Jr., Marques, Almeida, & Montilla, 2016; Telles, 2004). However, the country has experienced a consistent trend of reducing socioeconomic disparities for several decades. This movement has been interpreted with optimism in the field of public health in view of the expansion of community-based primary health care with improved vaccination coverage and reduced child mortality and undernutrition in children under five, among other important achievements (Szwarcwald et al., 2016; Victora et al., 2011).

Our findings show that more Indigenous children and adolescents died than their non-Indigenous counterparts, including those classified as black or brown, in both rural and urban residential settings. More specifically, the mortality curves presented here show that Indigenous children and adolescents were consistently at the most disadvantaged end of a marked gradient of ethnic-racial inequality in Brazil, despite important improvements to social and health equity at the national level. Mortality rates were also systematically higher than the rest of the Brazilian population independently of sex, age, and geopolitical region.

This huge gap separating Indigenous from non-Indigenous Brazilian mortality in children and adolescents exemplifies a more general picture of social, economic, and political inequities to which Indigenous peoples are exposed and which remained “invisible” until recently due to lack of data and low scientific and political priority afforded to Indigenous populations in national health research. Indigenous peoples’ health disparities associated with social inequities in Brazil result in a disproportionate burden of ill-health and suffering, producing an immediate and final outcome whereby approximately 25 children per 1,000 live births do not complete their first year of life. Furthermore, we can learn from the 2010 Brazilian national demographic census that those who survive face a childhood and adolescence characterized by marked social inequity and increased risk of mortality.

## Financial disclosure statement

This work was funded by the Wellcome Trust (grant 203486/Z/16/Z) and the Foundation for Scientific and Technological Development in Health (TED/FIOTEC 175/2018, project no. 25380.102279/2018-04) . The authors also thank the Brazilian National Council for Scientific and Technological Development (CNPq) for the concession of research fellowships for Coimbra, Queiroz, Santos, and Welch.

## CRediT authorship contribution statement

**Ricardo Ventura Santos:** Conceptualization, Funding acquisition, Investigation, Methodology, Writing - original draft, Writing - review & editing. **Gabriel Mendes Borges:** Conceptualization, Formal analysis, Investigation, Methodology, Software, Writing - original draft, Writing - review & editing. **Marden Barbosa de Campos:** Conceptualization, Formal analysis, Investigation, Methodology. **Bernardo Lanza Queiroz:** Conceptualization, Formal analysis, Investigation, Methodology, Writing - original draft, Writing - review & editing. **Carlos E.A. Coimbra:** Conceptualization, Investigation, Writing - original draft, Writing - review & editing. **James R. Welch:** Conceptualization, Investigation, Methodology, Writing - original draft, Writing - review & editing.

## Declaration of competing interest

We declare no competing interests. Study sponsors had no involvement in study design, data analysis, interpretation of data, writing of the report, or the decision to submit the paper for publication.
